# DecGPU: distributed error correction on massively parallel graphics processing units using CUDA and MPI

**DOI:** 10.1186/1471-2105-12-85

**Published:** 2011-03-29

**Authors:** Yongchao Liu, Bertil Schmidt, Douglas L Maskell

**Affiliations:** 1 School of Computer Engineering, Nanyang Technological University, 639798, Singapore

## Abstract

**Background:**

Next-generation sequencing technologies have led to the high-throughput production of sequence data (reads) at low cost. However, these reads are significantly shorter and more error-prone than conventional Sanger shotgun reads. This poses a challenge for the *de novo *assembly in terms of assembly quality and scalability for large-scale short read datasets.

**Results:**

We present DecGPU, the first parallel and distributed error correction algorithm for high-throughput short reads (HTSRs) using a hybrid combination of CUDA and MPI parallel programming models. DecGPU provides CPU-based and GPU-based versions, where the CPU-based version employs coarse-grained and fine-grained parallelism using the MPI and OpenMP parallel programming models, and the GPU-based version takes advantage of the CUDA and MPI parallel programming models and employs a hybrid CPU+GPU computing model to maximize the performance by overlapping the CPU and GPU computation. The distributed feature of our algorithm makes it feasible and flexible for the error correction of large-scale HTSR datasets. Using simulated and real datasets, our algorithm demonstrates superior performance, in terms of error correction quality and execution speed, to the existing error correction algorithms. Furthermore, when combined with Velvet and ABySS, the resulting DecGPU-Velvet and DecGPU-ABySS assemblers demonstrate the potential of our algorithm to improve *de novo *assembly quality for *de*-*Bruijn*-graph-based assemblers.

**Conclusions:**

DecGPU is publicly available open-source software, written in CUDA C++ and MPI. The experimental results suggest that DecGPU is an effective and feasible error correction algorithm to tackle the flood of short reads produced by next-generation sequencing technologies.

## Background

### Introduction

The ongoing revolution of next-generation sequencing (NGS) technologies has led to the production of high-throughput short read (HTSR) data (i.e. DNA sequences) at dramatically lower cost compared to conventional Sanger shotgun sequencing. However, the produced reads are significantly shorter and more error-prone. Additionally, *de novo *whole-genome shotgun fragment assemblers that have been optimized for Sanger reads, such as Altas [1], ARACHNE [2], Celera [3] and PCAP [4], do not scale well for HTSR data. Therefore, a new generation of *de novo *assemblers is required.

Several greedy short read assemblers, such as SSAKE [5], SHARCGS [6], VCAKE [7] and Taipan [8], have been developed based on *contig *extensions. However, these assemblers have difficulties in assembling repeat regions. The introduction of de Bruijn graphs for fragment assembly [9] has sparked new interests in using the de Bruijn graph approach for short read assembly. In the context of short read assembly, nodes of a de Bruijn graph represent all possible *k*-mers (a *k*-mer is a substring of length *k*), and edges represent suffix-prefix perfect overlaps of length *k*-1. Short read assemblers based on the de Bruijn graph approach include EULER-SR [10], Velvet [11], ALLPATHS [12], ABySS [13], and SOAPdenovo [14]. In a de Bruijn graph, each single-base error in a read induces up to *k *false nodes, and since each false node has a chance of linking to some other node, it is likely to induce false path convergence. Therefore, assembly quality of *de*-*Bruijn*-graph-based assemblers is expected to improve by detecting and fixing base errors in reads prior to assembly.

In addition to the error correction algorithms based on the spectral alignment problem (SAP) in [9] and [10], a new error correction algorithm called SHREC [15] has been proposed using a generalized suffix trie. Hybrid SHREC (hSHREC) [16] extends the work of SHREC by enabling the correction of substitutions, insertions, and deletions in a mixed set of short reads produced from different sequencing platforms. Unfortunately, due to the large size of NGS datasets, the error correction procedure before assembly is both time and memory consuming. Many-core GPU computing architectures have evolved rapidly and have already demonstrated their powerful compute capability to reduce the execution time of a range of demanding bioinformatics applications, such as protein sequence database search [17,18], multiple sequence alignment [19], and motif finding [20]. As a first step, Shi et al. [21] implemented CUDA-EC, a parallel error correction algorithm using NVIDIA's compute unified device architecture (CUDA), based on the SAP approach [9], where a Bloom filter data structure [22] is used to gain memory space efficiency. This algorithm has been further optimized by incorporating quality scores and filtration approach in [23]. However, the drawback of this approach is the assumption that the device memory of a single GPU is sufficient to store the genome information of the SAP, i.e. the spectrum *T*(*G*) (see Spectral alignment problem subsection). Thus, a distributed error correction approach is a good choice to further reduce execution time and to overcome memory constraints.

In this paper, we present DecGPU, the first parallel and distributed error correction algorithm for large-scale HTSR datasets using a hybrid combination of CUDA and message passing interface (MPI) [24] parallel programming models. DecGPU provides two versions: a CPU-based version and a GPU-based version. The CPU-based version employs coarse-grained and fine-grained parallelism using the MPI and Open Multi-Processing (OpenMP) [25] parallel programming models. The GPU-based version takes advantage of the CUDA and MPI parallel programming models and employs a hybrid CPU+GPU computing model to maximize the performance by overlapping the CPU and GPU computation. The distributed feature of our algorithm makes it a feasible and flexible solution to the error correction of large-scale HTSR datasets. Our algorithm is designed based on the SAP approach and uses a counting Bloom filter data structure [26] for memory space efficiency. Even though our algorithm also uses the filtration approach to reduce execution time like CUDA-EC, it has intrinsic differences from CUDA-EC, such as distributed *k*-mer spectrums, hybrid combination of different parallel programming models, and CUDA kernel implementations. Compared to the hSHREC algorithm, DecGPU shows superior error correction quality for both simulated and real datasets. As for the execution speed, on a workstation with two quad-core CPUs, our CPU-based version runs up to 22× faster than hSHREC. Furthermore, on a single GPU, the GPU-based version runs up to 2.8× faster than CUDA-EC (version 1.0.1). When combined with Velvet (version 1.0.17) and ABySS (version 1.2.1), the resulting DecGPU-Velvet and DecGPU-ABySS assemblers demonstrate the potential of our algorithm to improve *de novo *assembly quality for *de*-*Bruijn*-graph-based assemblers by correcting sequencing errors prior to assembly.

### Spectral alignment problem

The SAP approach detects and fixes base errors in a read based on the *k*-mer set *G*_*k *_of a genome *G*. Since the genome *G *is not known beforehand in a *de novo *sequencing project, SAP approximates *G*_*k *_using a *k*-mer spectrum *T*(*G*). *T*(*G*) is the set of all solid *k*-mers throughout all reads. A *k*-mer is called *solid *if its multiplicity throughout all reads is not less than a user-specified threshold *M*, and *weak *otherwise. If every *k*-mer in a read has an exact match in *T*(*G*), the read is called a *T-string*. Given an erroneous read *R*, SAP is defined to find a T-string R^* ^with minimal Hamming distance to *R*.

Two heuristics of SAP have been suggested: the iterative approach [9] and the dynamic programming approach [10]. The iterative approach attempts to transform weak *k*-mers in a read to solid ones by substituting some possibly erroneous bases through a voting algorithm. The dynamic programming approach attempts to find the shortest path that corresponds to a *T-string *with minimal edit distance. The underlying algorithm model of DecGPU is inspired by the iterative approach.

### Bloom filter data structure

The spectrum *T*(*G*) is the fundamental data structure for SAP-based error correction. For large-scale short read error correction, the major challenges posed by *T*(*G*) are the computational overhead for *k*-mer membership lookup and the memory constraint for *k*-mer storage. Hash tables are advantageous in execution time for membership lookup, but consume too much memory. Thus, we choose a Bloom filter, a very compact hash-based data structure, to achieve efficiency in terms of both lookup time and memory space. However, the space efficiency of a Bloom filter is gained by allowing false positive querying. The more elements inserted to the Bloom filter, the higher the probability of false positive querying. As such, a Bloom filter is more suitable for the cases where space resources are at a premium and a small number of false positives can be tolerated. Both conditions are met by our error correction algorithm, since false positives might only result in some unidentified sequencing errors.

A classical Bloom filter uses a bit array with *h *associated independent hash functions, supporting insertion and membership querying of elements. Initially, all buckets (1 bit per bucket) in a classical Bloom filter are set to zero. When inserting or querying an element, the *h *hash values of the element are first calculated using the *h *hash functions. When inserting an element, the corresponding buckets indexed by the hash values are set to 1. When querying an element, it returns the corresponding buckets. The element is likely to exist if all buckets are 1; and definitely does not exist, otherwise. The time for insertion and querying, of an element, is of constant time complexity, *O*(*h*), and is also independent of the number of inserted elements. The false positive probability (FPP) of a classical Bloom filter is calculated as(1)

where *N*_*B *_is the total number of buckets, *N*_*E *_is the number of elements, and *α *= *hN*_*E*_/*N*_*B*_.

To construct *T*(*G*), we need to record the multiplicity of each *k*-mer. However, because the classical Bloom filter does not store the number of *k*-mer occurrences, DecGPU instead chooses a counting Bloom filter to represent *T*(*G*). A counting Bloom filter extends a bucket of the classical Bloom filter from 1 bit to several bits. DecGPU uses 4 bits per bucket, supporting a maximum multiplicity of 15. When inserting an element, it increases (using saturation addition) the counter values of the corresponding buckets indexed by the hash values. When querying an element, it returns the minimum counter value of all the corresponding buckets, which is most likely to be the real multiplicity of the element. A counting Bloom filter has the same FPP as the corresponding classical Bloom filter.

### CUDA and MPI programming models

More than a software and hardware co-processing architecture, CUDA is also a parallel programming language extending general programming languages, such as C, C++ and Fortran with a minimalist set of abstractions for expressing parallelism. CUDA enables users to write parallel scalable programs for CUDA-enabled processors with familiar languages [27]. A CUDA program is comprised of two parts: a host program running one or more sequential threads on a host CPU, and one or more parallel *kernels *able to execute on Tesla [28] and Fermi [29] unified graphics and computing architectures.

A kernel is a sequential program launched on a set of lightweight concurrent threads. The parallel threads are organized into a grid of thread blocks, where all threads in a thread block can synchronize through barriers and communicate via a high-speed, per block shared memory (PBSM). This hierarchical organization of threads enables thread blocks to implement coarse-grained task and data parallelism and lightweight threads comprising a thread block to provide fine-grained thread-level parallelism. Threads from different thread blocks in the same grid are able to cooperate through atomic operations on global memory shared by all threads. To write efficient CUDA programs, it is important to understand the features of the different memory spaces, including non-cached global and local memory, cached texture and constant memory as well as on-chip PBSM and registers.

The CUDA-enabled processors are built around a fully programmable scalable processor array, organized into a number of streaming multiprocessors (SMs). For the Tesla architecture, each SM contains 8 scalar processors (SPs) and shares a fixed 16 KB of PBSM. For the Tesla series, the number of SMs per device varies from generation to generation. For the Fermi architecture, it contains 16 SMs with each SM having 32 SPs. Each SM in the Fermi architecture has a configurable PBSM size from the 64 KB on-chip memory. This on-chip memory can be configured as 48 KB of PBSM with 16 KB of L1 cache or as 16 KB of PBSM with 48 KB of L1 cache. When executing a thread block, both architectures split all the threads in the thread block into small groups of 32 parallel threads, called *warps*, which are scheduled in a single instruction, multiple thread (SIMT) fashion. Divergence of execution paths is allowed for threads in a warp, but SMs realize full efficiency and performance when all threads of a warp take the same execution path.

MPI is a *de facto *standard for developing portable parallel applications using the message passing mechanism. MPI works on both shared and distributed memory machines, offering a highly portable solution to parallel programming on a variety of machines and hardware topologies. In MPI, it defines each worker as a process and enables the processes to execute different programs. This multiple program, multiple data model offers more flexibility for data-shared or data-distributed parallel program design. Within a computation, processes communicate data by calling runtime library routines, specified for the C/C++ and Fortran programming languages, including point-to-point and collective communication routines. Point-to-point communication is used to send and receive messages between two named processes, suitable for local and unstructured communications. Collective (global) communication is used to perform commonly used global operations (e.g. reduction and broadcast operations).

## Implementation

### DecGPU error correction algorithm

**Figure 1:**
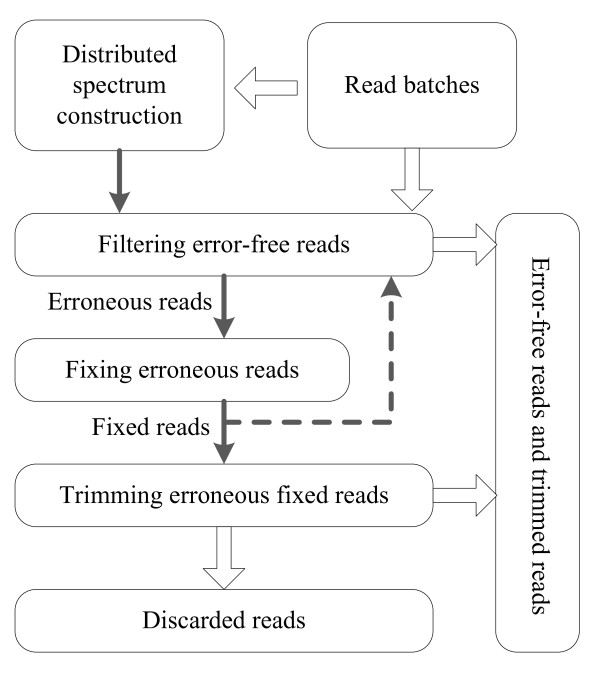
**Program workflow and data dependence between different stages**.

DecGPU consists of four major stages: (1) constructing the distributed *k*-mer spectrum, (2) filtering out error-free reads, (3) fixing erroneous reads using a voting algorithm, (4) trimming (or discarding entirely) the fixed reads that remain erroneous, and (5) an optional iterative policy between the filtering and fixing stages with intention to correct more than one base error in a single read. The second stage filters out error-free reads and passes down the remaining erroneous reads to the third stage. After the erroneous reads have been fixed, the fixed reads are either passed up to another filtering stage or down to the trimming stage, depending on whether the optional iterative policy is used. For a fixed read that remains erroneous, the trimming stage attempts to find the user-satisfied longest substring of the read, in which all *k*-mers are solid (the workflow and data dependence between stages are shown in Figure 1).

For DecGPU, a processing element (PE) *P*_*i *_refers to the *i*^th ^MPI process. Each MPI process has a one-to-one correspondence with a GPU device. Each *P*_*i *_therefore consists of two threads: a CPU thread and a GPU thread. This hybrid CPU+GPU computing model provides the potential to achieve performance maximization through the overlapping of CPU and GPU computation. The input reads of each stage are organized into batches to facilitate the overlapping. In the MPI runtime environment, DecGPU ensures the one-to-one correspondence between an MPI process and one GPU device by automatically assigning GPU devices to processes using a registration management approach. First, each process registers its hostname and the number of qualified GPU devices in its host to a specified master process. Secondly, the master process verifies the registrations by checking that, for a specific host, the number of GPU devices reported by all processes running on it must be the same and must not be less than the number of the processes. Finally, the master process enumerates each host and assigns a unique GPU device identifier to each process running on the host.

### Distributed spectrum construction

DecGPU distributes the *k*-mer spectrum that uses a counting Bloom filter. For the distributed spectrum, each *P*_*i *_holds a local spectrum *T*(*G*, *P*_*i*_) that is a subset of *T*(*G*). The set of all local spectrums {*T*(*G*, *P*_*i*_)} forms a partition of *T*(*G*); i.e. it holds:.(2)

where *N*_*PE *_is the number of PEs. DecGPU constructs the distributed spectrum by (nearly) evenly distributing the set of all possible *k*-mers (including their reverse complements) over all PEs. The location of a *k*-mer is determined using modular hashing. A *k*-mer is packed into an integer *I*_*k *_by mapping the bases {A, C, G, T} to the numerical values {0, 1, 2, 3}. The index of the PE that owns this *k*-mer is computed as *I*_*k *_% *N*_*PE*_. This distributed spectrum reduces the number of *k*-mers in a single spectrum by a factor of the number of PEs. Thus, we are able to keep an acceptable probability of false positives of *T*(*G*) with no need for a vast amount of device memory in a single GPU. Using this distributed spectrum, for the membership lookup of a *k*-mer, all PEs must simultaneously conduct the membership lookup of the *k*-mer in their local spectrums, and then perform collective operations to gain the final result.

**Figure 2:**
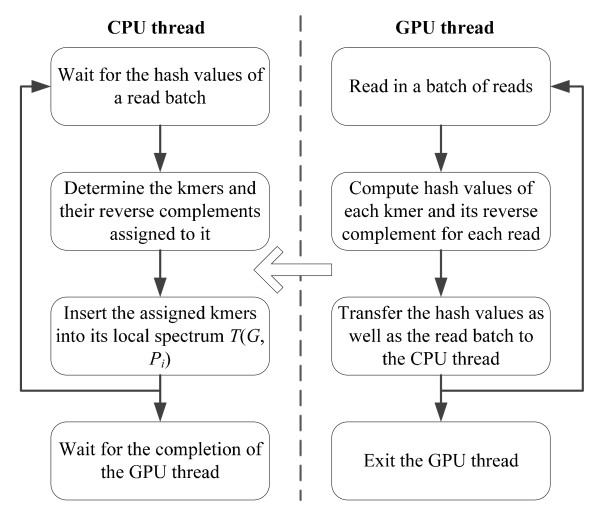
**Workflow of each PE for distributed spectrum construction**.

For the distributed spectrum construction, intuitively, the most effective approach is to allow each PE to build its local spectrum on its GPU device, where thousands of threads on the GPU device simultaneously calculate hash values of *k*-mers and determine their destinations. However, this approach requires the support for device-level global memory consistency or atomic functions, since different threads in the device might update the counter value at the same address in the counting Bloom filter. CUDA-enabled GPUs do not provide a mechanism to ensure device-level global memory consistency for all threads in a kernel when the kernel is running. CUDA does provide the support for atomic functions, but they are not byte-addressable. If using an integer for a bucket of a counting Bloom filter, the memory space efficiency of the Bloom filter will be significantly lost. In this case, we choose the CPU + GPU hybrid computing for the local spectrum construction of each *P*_*i *_(as shown in Figure 2). Since all input reads are organized into batches, each *P*_*i *_runs multiple iterations to complete the spectrum construction with each iteration processing a read batch. In each iteration, the CPU thread awaits the hash values of a read batch. When the hash values of a read batch are available, the CPU thread inserts *k*-mers, which are distributed to itself, into its local spectrum using the corresponding hash values. In the meantime, the GPU thread reads in another batch of reads, calculates the hash values for this batch, and then transfers the hash values as well as the read batch to the CPU thread.

Using CUDA, one read is mapped to one thread, where the thread computes the hash values of all *k*-mers and their reverse complements and determines their destination PEs in the read. All reads of a batch are stored in texture memory bound to linear memory. Because a *k*-mer is frequently accessed while calculating the hash values, the *k*-mer is loaded from texture memory to shared memory for improving performance. All the following stages store and access reads and *k*-mers in the same manner. A conversion table in constant memory is used for the conversion of a nucleotide base to its complement. The hash value arrays are allocated in global memory using the coalesced global memory allocation pattern [15].

### Filtering out error-free reads

The core of our distributed filtering algorithm is described as follows. For a specific read, each *P*_*i *_simultaneously checks in its local spectrum *T*(*G*, *P*_*i*_) the solidity of each *k*-mer of the read. Since each *k*-mer corresponds to a position in a read, *P*_*i *_uses a local solidity vector *SV*(*P*_*i*_) to record the *k*-mer existence for the read. If a *k*-mer belongs to *T*(*G*, *P*_*i*_), the corresponding position in *SV*(*P*_*i*_) is set to 0 and to 1 otherwise. After completing the solidity check of all *k*-mers, all PEs perform a logical AND reduction operation on the solidity vectors {*SV*(*P*_*i*_)} to gain the final global solidity vector *SV*. The read is error-free if all the positions in *SV *are 0 and erroneous otherwise. For each erroneous read, the values of *SV *are stored into a file, along with the read, for the future use of the fixing stage.

**Figure 3:**
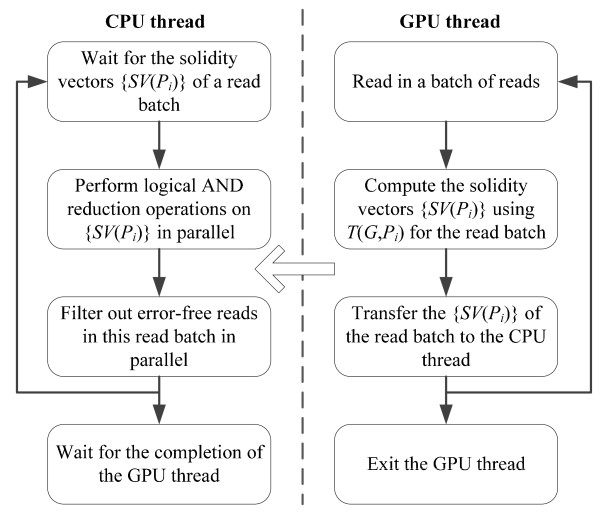
**Workflow of each PE for distributed error-free read filtering**.

Figure 3 shows the workflow of each PE for filtering out error-free reads. For each *P*_*i*_, the CPU thread receives the set {*SV*(*P*_*i*_)} of a read batch from the GPU thread, performs logical AND reduction operations on {*SV*(*P*_*i*_)} in parallel with the other PEs, and then processes the read batch in parallel with the other PEs to filter out error-free reads. Meanwhile, the GPU thread reads in a batch of reads, calculates {*SV*(*P*_*i*_)} of the batch using its local spectrum *T*(*G*, *P*_*i*_), and then transfers {*SV*(*P*_*i*_)} to the CPU thread. From this workflow, the calculation time of the solidity vectors on the GPUs does not scale with the number of PEs, but the execution time of the reduction operations and the error-free reads determination scales well with the number of PEs. Using CUDA, one read is mapped to one thread which builds the solidity vector of the read using *T*(*G*, *P*_*i*_). The solidity vectors are allocated in global memory in a coalesced pattern.

### Fixing erroneous reads

If a mutation error occurs at position *j *of a read of length *l*, this mutation creates up to *min*{*k*, *j*, *l*-*j*} erroneous *k*-mers that point to the same sequencing error. The aim of our fixing algorithm is to transform the *min*{*k*, *j*, *l*-*j*} weak *k*-mers to solid ones. In this case, a voting algorithm is applied to correct the most likely erroneous bases that result in these weak *k*-mers. The voting algorithm attempts to find the correct base by replacing all possible bases at each position of the *k*-mer and checking the solidities of the resulting *k*-mers.

**Figure 4:**
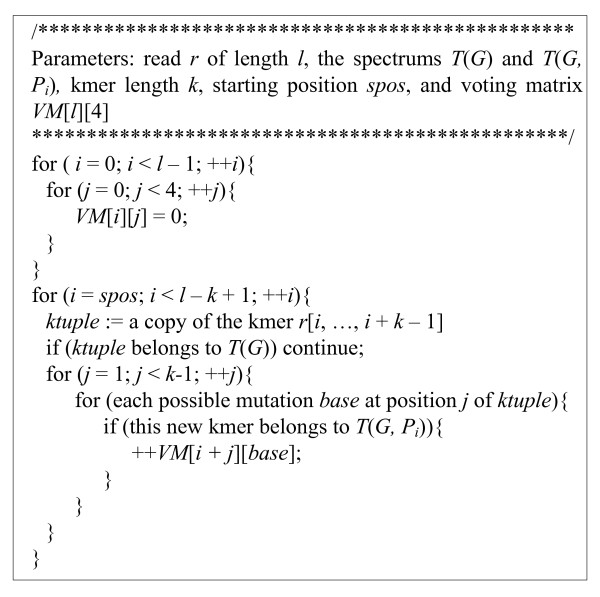
**Pseudocode of the CUDA kernel of the voting algorithm**.

The core of our distributed fixing algorithm is described as follows. For an erroneous read, each *P*_*i *_checks in *T*(*G*) the existence of all *k*-mers of the read from left to right. Because each *P*_*i *_does not hold a copy of *T*(*G*), the existence check in *T*(*G*) is conducted using the solidity vectors {*SV*} produced and saved by the filtering stage. If a *k*-mer does not belong to *T*(*G*), each *P*_*i *_invokes the voting algorithm to compute its local voting matrix *VM*(*P*_*i*_) using its local spectrum *T*(*G*, *P*_*i*_). After completing the voting matrix computation, all PEs perform an ADDITION reduction operation on the voting matrices {*VM*(*P*_*i*_)} to gain the final global voting matrix *VM *of the read. Then, a fixing procedure is performed using *VM *to correct the erroneous read. When enabling the optional iterative policy, for an erroneous read, a starting position *SPOS *is saved after completing the previous fixing iteration, which indicates that each *k*-mer starting before *SPOS *is solid in the read. In the current fixing iteration, the voting matrix computation starts from *SPOS*. Actually, after substituting an erroneous base with the voted (likely) correct base, we might introduce new errors even if there is really only one base error in a read. Hence, it is not necessarily the case that the more fixing iterations used, the more base errors that are corrected. Figure 4 shows the pseudocode of the CUDA kernel of the voting algorithm.

**Figure 5:**
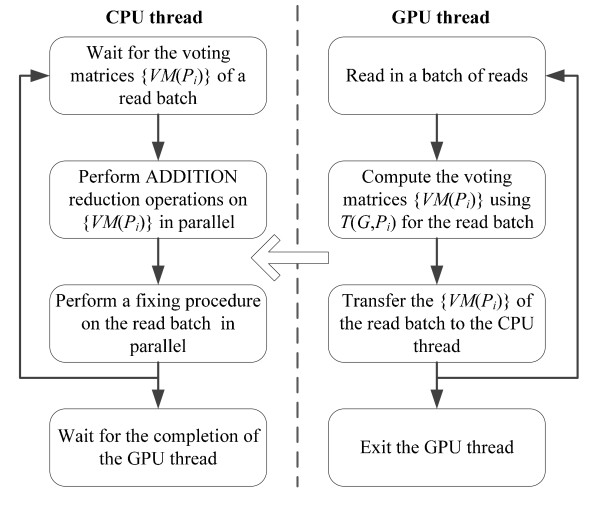
**Workflow of each PE for fixing erroneous reads**.

Figure 5 shows the workflow of each PE for fixing erroneous reads. For each *P*_*i*_, the CPU thread receives the voting matrices {*VM*(*P*_*i*_)} of a read batch from the GPU thread, performs ADDITION reduction operations on {*VM*(*P*_*i*_)} in parallel with the other PEs, and then fixes the erroneous reads in parallel with the other PEs. The GPU thread computes its local voting matrices {*VM*(*P*_*i*_)} of a read batch using *T*(*G*, *P*_*i*_), and then transfers the voting matrices to the CPU thread.

Using CUDA, one read is mapped to a thread which performs the voting algorithm on the read to gain the voting matrix. From Figure 4, the execution speed of the voting algorithm on GPUs highly depends on how frequently the threads in a warp diverge. The solidity vectors of the reads, used for checking *k*-mer existence in *T*(*G*), are stored in texture memory bound to linear memory. The voting matrices are allocated in global memory in a coalesced pattern.

### Trimming erroneous reads

After fixing errors in erroneous reads, some reads are still not *T-strings*. In this case, a trimming procedure is performed on the fixed reads that remain erroneous. For an erroneous read, all PEs cooperate to compute the solidity vector *SV *of the read using the same algorithm as in the filtering stage. After gaining *SV*, the algorithm attempts to find the user-satisfied longest substring of the read, in which all *k*-mers are solid. The read is trimmed if such a substring is found and discarded entirely, otherwise. Each *P*_*i *_runs the same workflow as in the filtering stage, except that after gaining the solidity vectors {*SV*} of a read batch, the CPU thread performs the trimming procedure in parallel with the other PEs, instead.

## Results

We have evaluated the performance of DecGPU from three perspectives: (1) the error correction quality both on simulated and real short read datasets; (2) *de novo *assembly quality improvement after combining our algorithm with Velvet (version 1.0.17) and ABySS (version 1.2.1); and (3) the scalability with respect to different number of compute resources for the CPU-based and GPU-based versions respectively. Six simulated short read datasets (the first six datasets in Table 1) and three real Illumina GA short read datasets (the last three datasets in Table 1, named after their accession numbers in NCBI Sequence Read Archive [30]) are used to measure the accuracy of correction and the *de novo *assembly quality. For the six simulated datasets, they are simulated from the E. coli K12 MG1665 reference genome (NC_000913) with different read lengths, coverage and error rates. For the three real datasets, the SRR001665 dataset is a paired-end dataset and the other two are single-end. The SRR001665 dataset consists of about 20.8 million paired-end 36-basepair (bp) reads generated from a 200-bp insert size of an E. coli library (SRX000429), and has been used in [13] and [14] to assess the assembly qualities of various assemblers.

**Table 1:** Simulated and real short read datasets

Datasets	Read length	Coverage	Error rate	No. of Reads
D30X1.5	36	30	1.5%	3866000

D30X3.0	36	30	3.0%	3860000

D75X1.5	36	75	1.5%	9666000

D75X3.0	36	75	3.0%	9666000

D150X1.5	72	150	1.5%	9666000

D150X3.0	72	150	3.0%	9666000

SRR006331	36	69	-	1693848

SRR016146	51	81	-	4438066

SRR001665	36	162	-	20816448

All the following tests are conducted on a workstation computer and a computing cluster with eight compute nodes that are connected by a high-speed Infiniband switch. The workstation computer has two quad-core Intel Xeon E5506 2.13 GHz processors and 16 GB RAM running the Linux operating system (OS). For the computing cluster, each compute node consists of an AMD Opteron 2378 quad-core 2.4 GHz processor and 8 GB RAM running the Linux OS with the MVAPICH2 library [31]. Furthermore, two Tesla S1070 quad-GPU computing systems are installed and connected to four nodes of the cluster. A single Tesla T10 GPU of a Tesla S1070 system consists of 30 SMs comprising 240 SPs and 4 GB RAM. If not specified, for all the following tests, DecGPU uses the default parameters (i.e. the *k*-mer length is set to 21, the multiplicity threshold *M *to 6, the maximum allowable number of bases to be trimmed to 4, and one fixing iteration), and hSHREC sets the strictness value to 5 for the first four simulated datasets and 6 for the last two simulated datasets, using eight threads.

We have evaluated the performance of our algorithm using the simulated datasets in terms of: (1) the ability to detect reads as error-free or erroneous, and (2) the ability to correct erroneous reads. The detection of erroneous reads is a binary classification test, where an input read is classified into either the error-free group or the erroneous group. Table 2 shows the corresponding definitions of true positive (TP), false positive (FP), true negative (TN) and false negative (FN). The sensitivity and specificity measures are defined as(3)(4)

**Table 2:** Definitions for the read binary classification test

Classification	Read Condition	
	
	Erroneous	Error-free
Detected as erroneous	TP	FP

Detected as error-free	FN	TN

The results of the classification test are shown in Table 3 for the six simulated datasets, where the sensitivity and specificity values have been multiplied by 100. From the sensitivity measure, DecGPU and hSHREC achieve comparable performance for all datasets, where the sensitivity is > 99.80% for each dataset, meaning that only very few erroneous reads remain undetected. However, as for the specificity measure, the performance of hSHREC degrades very fast with the increase of dataset size and coverage. For each of the last four simulated datasets, the specificity of DecGPU is > 99.80%, clearly outperforming hSHREC. For the two low-coverage D30X1.5 and D30X3.0 datasets, DecGPU gives poorer specificity than hSHREC. However, after setting the multiplicity threshold *M *to 3 and 2, instead of the default 6, DecGPU yields a specificity of 99.52% and 99.32% for the two datasets respectively, better than hSHREC.

**Table 3:** Summary of the classification test for simulated datasets

Datasets	Algorithm	TP	FP	FN	TN	Sensitivity	Specificity
D30X1.5	DecGPU	1620660	349908	253	1895179	99.98	84.41
	
	hSHREC	1617685	13998	3228	2231089	99.80	99.38

D30X3.0	DecGPU	2575411	660533	306	629750	99.99	48.81
	
	hSHREC	2571520	31367	4197	1258916	99.84	97.57

D75X1.5	DecGPU	4053688	23	1024	5611265	99.97	100.00
	
	hSHREC	4053827	4990124	885	621164	99.98	11.07

D75X3.0	DecGPU	6435328	3481	1621	3225570	99.97	99.89
	
	hSHREC	6436305	3129803	644	99248	99.99	3.07

D150X1.5	DecGPU	6406078	2	5395	3254525	99.92	100.00
	
	hSHREC	6411346	3185858	127	68669	100.00	2.11

D150X3.0	DecGPU	8578176	1	8651	1079172	99.90	100.00
	
	hSHREC	8586743	1056392	84	22781	100.00	2.11

The performance of correcting erroneous reads is evaluated using the simulated datasets from two aspects. The first aspect is to compare the error rates before and after error correction. The error rates are calculated by doing a base-by-base comparison with their respective original reads (without errors). It is possible that a corrected read does not have the same length with its original read. In this case, the shorter read is mapped with no gaps to the longer one by iteratively changing the starting positions. We choose the mapping with the minimal number of base errors, and then add the number of bases in the shorter one to the total number of bases for the future calculation of error rates. For DecGPU, we vary the number of fixing iterations with the intention to find and correct more than one erroneous base in a single read. We have compared the accuracy and execution time of DecGPU to hSHREC (see Table 4) on the above workstation with eight CPU cores. Table 4 shows that DecGPU significantly reduces the error rates of all datasets (particularly reducing the error rate of D75X1.5 from 1.500% to 0.248% and the error rate of D75X3.0 from 3.000% to 0.988%), clearly outperforming hSHREC. Furthermore, on the dual quad-core workstation, the CPU-based DecGPU version runs up to 22× faster when performing one fixing iteration and up to 19× faster when performing two fixing iterations compared to hSHREC. For DecGPU, the error rates are further reduced for all datasets when using two fixing iterations instead of only one. However, we found that a further increase of iterations does not significantly reduce the error rates further. As for the execution time, the second fixing iteration does not result in a large execution time increase, since it only corrects the remaining erroneous reads.

**Table 4:** The error rates and execution time comparison for DecGPU and Hybrid SHREC

Datasets	Original Error Rate (%)	Corrected Error Rate (%)			Time (seconds)		
		
		DecGPU		hSHREC	DecGPU		hSHREC
					
		one fixing	two fixing		one fixing	two fixing	
D30X1.5	1.498	0.426	0.341	0.713	125	145	2721

D30X3.0	3.003	1.773	1.625	2.014	164	217	2882

D75X1.5	1.500	0.347	0.248	3.936	288	348	4380

D75X3.0	3.000	1.262	0.988	4.058	375	473	5079

D150X1.5	1.500	0.579	0.348	3.233	981	1118	11047

D150X3.0	3.001	1.781	1.241	4.082	1254	1489	12951

The second aspect is to evaluate the correct correction rate, incorrect correction rate, and the rate of newly introduced errors, relative to the total number of original base errors. When performing error correction, correction operations will result in the following four cases:

• *Correct Corrections *(*CC*): meaning that original erroneous bases have been changed to the correct ones;

• *Incorrect Corrections *(*IC*): meaning that original erroneous bases have been changed to other wrong ones;

• *Errors Unchanged *(*EU*): meaning that original erroneous bases remain the same;

• *Errors Introduced *(*EI*): meaning that original correct bases have been changed to be incorrect, thus introducing new base errors.

In this paper, we define three measures relative to the total number of original base errors: correct correction rate *R*_*CC*_, incorrect correction rate *R*_*IC*_, and correction error rate *R*_*EI*_, to facilitate the error correction accuracy comparison. *R*_*CC *_indicates the proportion of the original erroneous bases that have been corrected, *R*_*EI *_indicates the proportion of the original erroneous bases that have been changed to other wrong bases, and *R*_*EI *_indicates the ratio of the original correct bases that have been changed to be incorrect. For *R*_*CC*_, the larger value means the better performance, and for *R*_*IC *_and *R*_*EI*_, the smaller value the better performance. The *R*_*CC*_, *R*_*IC *_and *R*_*EI *_measures are calculated as(5)(6)(7)

In this test, for DecGPU, we do not trim the fixed reads that remain erroneous, and use two fixing iterations. For hSHREC, we only use the reads that have the same lengths with their original reads after correction, because the correspondence relationship between bases is difficult to be determined for two reads of different lengths. Table 5 shows the performance comparison in terms of the three measures between DecGPU and hSHREC, where the value of *R*_*CC*_, *R*_*IC *_and *R*_*EI *_has been multiplied by 100. For *R*_*CC*_, hSHREC yields better performance for the first three datasets and DecGPU performs better for the last three datasets. However, hSHREC degrades very rapidly (down to 5.73%) with the increase of coverage and original error rate, while DecGPU remains relatively consistent. For *R*_*IC *_and *R*_*EI*_, DecGPU clearly outperforms hSHREC for each dataset, where DecGPU miscorrected ≤ 0.04% bases and introduced ≤ 0.08% new base errors, but hSHREC miscorrected ≥ 0.30% (up to 0.73%) bases, and introduced ≥ 6.95% (up to 47.67%) new base errors.

**Table 5:** Performance comparison with respect to *R_C__C_*, *R_I__C _*and *R_E__I _*measures

Datasets	Algorithms	CC	IC	EU	EI	**R**_**CC**_	**R**_**IC**_	**R**_**EI**_
D30X1.5	DecGPU	1275967	191	809207	893	61.19	0.01	0.05
	
	hSHREC	1736112	10960	214851	125381	88.49	0.56	6.95

D30X3.0	DecGPU	1611459	344	2567906	2932	38.55	0.01	0.08
	
	hSHREC	2983112	27448	764097	326466	79.03	0.73	9.38

D75X1.5	DecGPU	3373714	388	1844213	530	64.65	0.01	0.02
	
	hSHREC	1431267	27988	3256061	2219648	30.35	0.59	47.67

D75X3.0	DecGPU	5425615	746	5013497	1122	51.97	0.01	0.02
	
	hSHREC	757454	29924	9248234	1250738	7.55	0.30	12.76

D150X1.5	DecGPU	7242425	2913	3196883	1004	69.36	0.03	0.04
	
	hSHREC	741722	37618	9034830	3345778	7.56	0.38	34.47

D150X3.0	DecGPU	11221669	7593	9655700	2121	53.73	0.04	0.05
	
	hSHREC	1152718	71504	18896523	3136637	5.73	0.36	15.94

**Figure 6:**
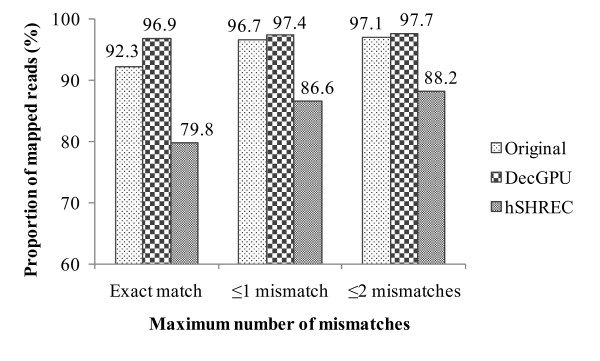
**Percentage of mapped reads as a function of maximum number of mismatches**.

Furthermore, we have measured the error correction quality of DecGPU in terms of mapped reads after aligning the reads to their reference genome. We vary the maximum allowable number of mismatches in a single read (or seed) to see the proportion changes. The SRR001665 dataset and Bowtie (version 0.12.7) [32] short read alignment algorithm are used for the evaluation. For Bowtie, the default parameters are used except for the maximum allowable number of mismatches, and for hSHREC, we have set the strictness value to 7. The proportion of mapped reads is calculated in three cases: exact match, ≤ one mismatch, and ≤ two mismatches (see Figure 6). After error correction with DecGPU, the proportion of mapped reads is higher than the original reads in each case. However, after error correction with hSHREC, the proportion for each dataset goes down in each case. This might be caused by the fact that some reads become very short after error correction with hSHREC.

Error correction prior to assembly is important for short read assemblers based on the *de Brujin *graph approach. To demonstrate how our algorithm affects *de novo *assembly quality, we have assessed the assembly quality before and after using our algorithm to correct errors for two popular assemblers: Velvet (version 1.0.17) and ABySS (version 1.2.1). Both assemblers do not internally incorporate error correction prior to assembly. We have carefully tuned the parameters with the intention to gain the highest assembly quality for the stand-alone Velvet and ABySS assemblers. We compared the assemblers in terms of N50, N90 and maximum *contig *or *scaffold *sizes using the three real datasets. The N50 (N90) *contig *or *scaffold *size is calculated by ordering all assembled sequences by length, and then adding the lengths from the largest to the smallest until the summed length exceeds 50% (90%) of the reference genome size. For these calculations, we use the reference genome sizes of 877438, 2801838, and 4639675 for the datasets SRR006331, SRR016146 and SRR001665 respectively. For the calculation of *scaffold *sizes, the intra-scaffold gaps are included. To see the difference in assembly quality before and after error correction, we use the same set of parameters with the stand-alone assemblers for our resulting DecGPU-Velvet (D-Velvet) and DecGPU-ABySS (D-ABySS) assemblers to conduct the assembly work (assembly results are shown in Table 6), where DecGPU uses two fixing iterations. From Table 6, D-Velvet yields superior N50 *contig *sizes to Velvet, with not always higher N90 and maximum *contig *sizes, for all datasets. D-ABySS gives comparable N50, N90 and maximum *contig *sizes with ABySS for all datasets. When scaffolding the paired-end SRR001665, D-ABySS produces larger N50 *scaffold *size than ABySS, but D-Velvet failed to outperform Velvet. However, after further tuning the assembly parameters, D-Velvet yields superior N50 *scaffold *size to Velvet for SRR001665 (see Table 7). Moreover, larger N50 *contig *sizes are produced by D-ABySS on SRR006331 and SRR016146 respectively, which are better than the outcome of ABySS. All these results suggest that our algorithm has the potential to improve the *de novo *assembly quality for *de*-*Bruijn*-graph-based assemblers. The number of assembled sequences ("#Seq" column in Tables 6 and 7) only counts in the sequences of lengths ≥ 100 bps, and the assembly output can be obtained from Additional file 1.

**Table 6:** Assembly quality and parameters for different assemblers

Datasets	Type	Assembler	N50	N90	MAX	#Seq	Parameters
SRR006331	*Contig*	Velvet	6229	1830	21166	288	k = 23, cov_cutoff = auto
			
		D-Velvet	7411	1549	17986	282	
		
		ABySS	5644	1505	15951	334	k = 24
			
		D-ABySS	4789	1216	12090	371	

SRR016146	*Contig*	Velvet	34052	7754	112041	301	k = 31, cov_cutoff = auto
			
		D-Velvet	34898	7754	134258	292	
		
		ABySS	34124	7758	112038	297	k = 33
			
		D-ABySS	34889	7916	134314	297	

SRR001665	*Contig*	Velvet	17900	4362	73058	601	k = 29, cov_cutoff = auto
			
		D-Velvet	18484	4687	73058	586	
		
		ABySS	18161	4364	71243	603	k = 30
			
		D-ABySS	18161	4604	73060	595	
	
	*Scaffold*	Velvet	95486	26570	268283	179	k = 31,exp_cov = auto, cov_cutoff = auto
			
		D-Velvet	95429	26570	268084	175	
		
		ABySS	96308	25780	268372	124	k = 33, n = 10
			
		D-ABySS	96904	27002	210775	122	

**Table 7:** Assembly quality and parameters after further tuning parameters for some datasets

Datasets	Type	Assembler	N50	N90	MAX	#Seq	Parameters
SRR006331	*Contig*	D-ABySS	6130	1513	16397	311	k = 24, c = 7

SRR001665	*Contig*	D-ABySS	20068	5147	73062	565	k = 31, c = 12
	
	*Scaffold*	D-Velvet	101245	30793	269944	146	k = 31, exp_cov = 36, cov_cutoff = 13

**Figure 7:**
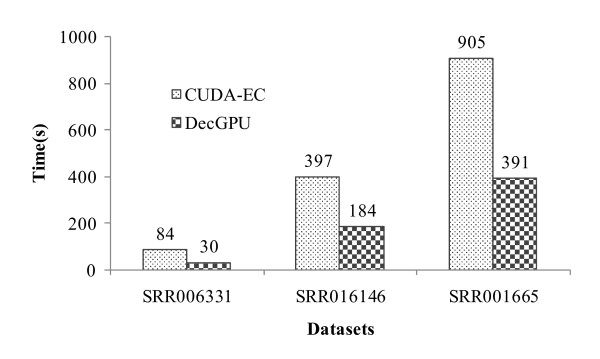
**Execution time comparison between DecGPU and CUDA-EC**.

The execution speed of DecGPU is evaluated using the three real datasets in terms of: (1) scalability of the CPU-based and GPU-based versions with respect to different number of compute resources, and (2) execution time of the GPU-based version compared to that of CUDA-EC (version 1.0.1) on a single GPU. Both of the assessments are conducted on the already described computing cluster. In addition to the absolute execution time, we use another measure, called Million Bases Processed per Second (MBPS), to indicate execution speed and make the evaluation more independent of datasets. Table 8 gives the execution time (in seconds) and MBPS of the two versions on different number of CPU cores and different number of GPUs respectively. On a quad-core CPU, DecGPU achieves a performance of up to 1.7 MBPS for the spectrum construction ("Spectrum" row in the table) and up to 2.8 MBPS for the error correction part ("EC" row in the table). On a single GPU, our algorithm produces a performance of up to 2.9 MBPS for the spectrum construction and up to 8.0 MBPS for the error correction part. However, it can also be seen that our algorithm does not show good runtime scalability with respect to the number of compute resources for either version. This is because our algorithm intends to solve the memory constraint problem for large-scale HTSR datasets, i.e. it requires the combination of results from distributed spectrums through collective reduction operations on all reads, limiting its runtime scalability. Subsequently, we compared the execution speed of our algorithm with that of CUDA-EC on a single Tesla T10 GPU (see Figure 7), where CUDA-EC sets *k*-mer length to 21 and the minimum multiplicity to 5. DecGPU runs on average about 2.4× faster than CUDA-EC, with a highest of about 2.8 ×.

**Table 8:** Execution time and MBPS of DecGPU on different number of compute resources

Datasets			No. of CPU cores				No. of GPUs			
			
			4	8	16	32	1	2	4	8
SRR006331	Spectrum	Time(s)	36	19	11	7	21	15	9	9
		
		MBPS	1.7	3.2	5.5	8.7	2.9	4.1	6.8	6.8
	
	EC	Time(s)	35	38	41	42	9	11	18	23
		
		MBPS	1.7	1.6	1.5	1.5	6.8	5.5	3.4	2.7

SRR016146	Spectrum	Time(s)	194	96	51	30	121	86	46	48
		
		MBPS	1.2	2.4	4.4	7.5	1.9	2.6	4.9	4.7
	
	EC	Time(s)	194	168	175	206	63	53	43	45
		
		MBPS	1.2	1.3	1.3	1.1	3.6	4.3	5.3	5.0

SRR001665	Spectrum	Time(s)	473	247	136	86	297	231	133	137
		
		MBPS	1.6	3.0	5.5	8.7	2.5	3.2	5.6	5.5
	
	EC	Time(s)	266	223	251	306	94	85	85	99
		
		MBPS	2.8	3.4	3.0	2.4	8.0	8.8	8.8	7.6

As mentioned above, DecGPU achieves memory efficiency through the use of a counting Bloom filter. From Equation 1, the FPP of a counting Bloom filter depends on the values *h *and *α*. DecGPU uses eight hash functions (i.e. *h *= 8) and has a maximal *N*_*B *_of 2^32^. Thus, for specific values of *α *and FPP, we can calculate the maximal value of *N*_*E*_. Table 9 shows the FPP and the maximal *N*_*E *_for a counting Bloom filter for some representative values of *α*. In the following, we will discuss how to estimate the maximal size of a short read dataset that can be processed with a fixed FPP by *N*_*PE *_MPI processes (i.e. we are using *N*_*PE *_counting Bloom filters on *N*_*PE *_compute nodes). Following [11], the expected number of times a unique *k*-mer in a genome is observed in a short read dataset with coverage *C *and read length *L *can be estimated as(8)

**Table 9:** FPP and maximal *N*_*E *_for representative *α *value

*α*	FPP	**Maximal *N***_***E***_
1	2.5 × 10^-2^	536870912

0.5	5.7 × 10^-4^	268435456

0.25	5.7 × 10^-6^	134217728

0.125	3.6 × 10^-8^	67108864

Thus, the number of reads *N*_*R *_in the dataset, which can be processed with a fixed FPP by *N*_*PE *_MPI processes, can be estimated as(9)

From Equation 9, we can see that *N*_*R *_is directly proportional to *N*_*PE*_; i.e. the maximal number of reads scales linearly with the number of compute nodes. Next, we use an example to illustrate how the memory consumption of our algorithm scales with the number of reads. For an example dataset with *C *= 75 and *L *= 36, when *N*_*PE *_= 8, the maximal *N*_*R *_is estimated as 2.24 billion (80.5 billion bases) for *α *= 0.25 and as 4.47 billion (161.1 billion bases) for *α *= 0.5. Because each bucket takes 4 bits and the maximal *N*_*B *_is 2^32^, the peak memory consumption of a counting Bloom filter is 2 GB. Hence, the maximal total memory consumption is only 2 GB × *N*_*PE *_= 16 GB for such large a dataset. DecGPU uses *α *= 0.25 by default.

The above observations and discussions demonstrate that DecGPU has superior capabilities in both error correction quality and execution speed compared to existing error correction algorithms. Even though our algorithm does not show good parallel scalability with respect to different number of computing resources, the distributed feature of our algorithm does provide a feasible and flexible solution to the error correction of large-scale HTSR datasets.

## Conclusions

In this paper, we have presented DecGPU, the first parallel and distributed error correction algorithm for large-scale HTSR using a hybrid combination of CUDA and MPI parallel programming models. Our algorithm is designed based on the SAP approach and uses a counting Bloom filter data structure to gain space efficiency. DecGPU provides two versions: a CPU-based version and a GPU-based version. The CPU-based version employs coarse-grained and fine-grained parallelism using MPI and OpenMP parallel programming models. The GPU-based version takes advantage of the CUDA and MPI programming models, and employs a hybrid CPU+GPU computing model to maximize the performance by overlapping the CPU and GPU computation. Compared to hSHREC, our algorithm shows superior error correction quality for both simulated and real datasets. On a workstation with two quad-core CPUs, our CPU-based version runs up to 22× faster than hSHREC. On a single GPU, the GPU-based version runs up to 2.8× faster than CUDA-EC. Furthermore, the resultant D-Velvet and D-ABySS assemblers demonstrate that our algorithm has the potential to improve *de novo *assembly quality, through prior-assembly error correction, for *de*-*Bruijn*-graph-based assemblers. Although our algorithm does not show good parallel runtime scalability with respect to the number of computing resources, the distributed characteristic of DecGPU provides a feasible and flexible solution to solve the memory scalability problem for error correction of large-scale datasets.

## Availability and requirements

• **Project name**: DecGPU

• **Project home page**: http://decgpu.sourceforge.net

• **Operating system**: 64-bit Linux

• **Programming language**: C++, CUDA, and MPI 2.0

• **Other requirements**: CUDA SDK and Toolkits 2.0 or higher

• **Licence**: GNU General Public License (GPL) version 3

## List of abbreviations

CPU: Central Processing Unit; CUDA: Compute Unified Device Architecture; FPP: False Positive Probability; GPU: Graphics Processing Units; HTSR: High-Throughput Short Reads; MBPS: Million Bases Processed per Second; MPI: Message Passing Interface; NGS: Next-Generation Sequencing; OpenMP: Open Multi-Processing; OS: Operating System; PBSM: Per-Block Shared Memory; SAP: Spectral Alignment Problem; SIMT: Single Instruction, Multiple Thread; SM: Streaming Multiprocessor; SP: Scalable Processor; PE: Processing Element.

## Authors' contributions

YL conceptualized the study, carried out the design and implementation of the algorithm, performed benchmark tests, analyzed the results and drafted the manuscript; BS conceptualized the study, participated in the algorithm optimization and analysis of the results and contributed to the revising of the manuscript; DLM conceptualized the study, participated in the analysis of the results, and contributed to the revising of the manuscript. All authors read and approved the final manuscript.

## Supplementary Material

Additional file 1 **Assembled sequences of different assemblers**. This file contains the assembled sequences (*contigs *or *scaffolds*) for the assemblers Velvet, ABySS, DecGPU-Velvet and DecGPU-ABySS for the three real datasets. Click here for file
