# Supplementary material for: DecGPU: distributed error correction on massively parallel graphics processing units using CUDA and MPI
Source: BMC Bioinformatics. 2011 Mar 29;12:85. doi: 10.1186/1471-2105-12-85 (PMC3072957; doi:10.1186/1471-2105-12-85)
Supplement: Additional file 1 — Assembled sequences of different assemblers. This file contains the assembled sequences (contigs or scaffolds) for the assemblers Velvet, ABySS, DecGPU-Velvet and DecGPU-ABySS for the three real datasets. [file 1471-2105-12-85-S1.ZIP › DecGPU_Additional_file_1/AssemblyOutput.doc]

There are two folders in this package: Outcome(I) and Outcome(II).

- Outcome(I) folder contains the assembled sequences (*contigs* or *scaffolds*) for Velvet (version 1.0.17) , DecGPU-Velvet, ABySS (version 1.2.1) and DecGPU-ABySS, where the corresponding assemblers use the same set of parameters. These set of parameters are obtained by tuning the stand-alone assembler Velvet (or ABySS) to produce the best assembly quality.
- Outcome(2) folder contains the assembled sequences for DecGPU-Velvet and DecGPU-ABySS, after further tuning the assembly parameters.

In each folder, there are several sub-folders, named after the name of the assembler used to produce the assembled sequences in them. In any sub-folder, a file name XXXX-contigs.fa means that it contains the *contigs* for single-end or paired-end dataset XXXX, and a file name YYYY-scaffolds.fa means that it contains *scaffolds* for paired-end dataset YYYY. For example, in the Velvet sub-folder of Outcome(I), the file SRR006331-contigs.fa contains the *contigs* of dataset SRR006331 assembled by Velvet, and the file SRR001665-scaffolds.fa contains the *scaffolds* of dataset SRR001665 assembled by Velvet.
